# Structure of Blood Coagulation Factor VIII in Complex With an Anti-C2 Domain Non-Classical, Pathogenic Antibody Inhibitor

**DOI:** 10.3389/fimmu.2021.697602

**Published:** 2021-06-10

**Authors:** Estelle K. Ronayne, Shaun C. Peters, Joseph S. Gish, Celena Wilson, H. Trent Spencer, Christopher B. Doering, Pete Lollar, P. Clint Spiegel, Kenneth C. Childers

**Affiliations:** ^1^ Department of Chemistry, Western Washington University, Bellingham, WA, United States; ^2^ Department of Pediatrics, Aflac Cancer and Blood Disorders Center, Children’s Healthcare of Atlanta, Emory University, Atlanta, GA, United States

**Keywords:** factor VIII, blood coagulation, x-ray crystallography, antibody inhibitors, antibody binding

## Abstract

Factor VIII (fVIII) is a procoagulant protein that binds to activated factor IX (fIXa) on platelet surfaces to form the intrinsic tenase complex. Due to the high immunogenicity of fVIII, generation of antibody inhibitors is a common occurrence in patients during hemophilia A treatment and spontaneously occurs in acquired hemophilia A patients. Non-classical antibody inhibitors, which block fVIII activation by thrombin and formation of the tenase complex, are the most common anti-C2 domain pathogenic inhibitors in hemophilia A murine models and have been identified in patient plasmas. In this study, we report on the X-ray crystal structure of a B domain-deleted bioengineered fVIII bound to the non-classical antibody inhibitor, G99. While binding to G99 does not disrupt the overall domain architecture of fVIII, the C2 domain undergoes an ~8 Å translocation that is concomitant with breaking multiple domain-domain interactions. Analysis of normalized B-factor values revealed several solvent-exposed loops in the C1 and C2 domains which experience a decrease in thermal motion in the presence of inhibitory antibodies. These results enhance our understanding on the structural nature of binding non-classical inhibitors and provide a structural dynamics-based rationale for cooperativity between anti-C1 and anti-C2 domain inhibitors.

## Introduction

Hemophilia A is an X-linked recessive disorder that is caused by mutation to coagulation factor VIII (fVIII). Patients with hemophilia A are prone to uncontrolled bleeding events and require regular infusions of recombinant or plasma-derived fVIII to maintain functional coagulation (1, 2). In approximately 30% of hemophilia A treatment cases, patients will produce antibodies that inhibit infused fVIII and reduce treatment efficacy (3, 4). Furthermore, acquired hemophilia A can develop in healthy individuals through an autoimmune response, producing antibody inhibitors which bind to and inhibit the cofactor activity of native fVIII (5). Inhibitory activity is detected and quantitated by the Bethesda assay of neutralization of fVIII coagulant activity *in vitro* (6). Immune tolerance induction has been demonstrated to overwhelm the immune system through frequent, high-dosages of fVIII with modest success (3), but can be a physical and financial burden for the patient (7).

Coagulation fVIII is a multidomain glycoprotein that circulates in the bloodstream as a heterodimer of the heavy chain (A1-A2) and light chain (A3-C1-C2) while bound to von Willebrand factor (vWf) to prevent premature clearance and/or degradation (2, 8). Once cleaved by thrombin, activated fVIII (fVIIIa) dissociates from vWf and binds activated platelet surfaces, likely through embedding several solvent-exposed hydrophobic loops on the C1 and C2 domains (9–11). Binding of fVIIIa to activated factor IX (fIXa), a serine protease, forms the ‘intrinsic’ tenase complex, which amplifies the generation of activated factor X (fXa) and subsequently thrombin.

Previous studies indicate the A2, C1, and C2 domains to be highly immunogenic (4, 12–15). Anti-C2 domain inhibitors represent a diverse group of fVIII neutralizing antibodies and are categorized as classical and non-classical antibodies (16–18). Classical antibodies inhibit fVIII binding to vWf and platelet surfaces and their associated epitopes are categorized into groups A, AB or B (16). X-ray crystal structures of the isolated C2 domain bound classical inhibitors such as BO2C11 (19) and 3E6 (20, 21) have identified unique conformational epitopes and demonstrated how inhibitor binding reduces circulatory levels of fVIII. Conversely, non-classical antibodies prevent fVIII activation by thrombin or fXa and their epitopes are categorized into groups BC and C (16). Non-classical antibodies are the most common pathogenic inhibitors in hemophilia A murine models (16) and inhibitors with overlapping epitopes have been detected in hemophilia A patient plasma (17). Group BC inhibitors are the most common anti-C2 antibodies and display above-average titer levels, particularly in patients with acquired hemophilia A (16), representing a significant clinical complication.

The pathogenic, non-classical murine monoclonal antibody inhibitor G99 is a group BC inhibitor with specific inhibitory activity of 15,000 Bethesda units per mg IgG (16). The G99 inhibitor binds to several solvent-exposed loops in the C2 domain that are predicted to interact with thrombin and fIXa (22, 23). Epitope mapping based on hydrogen-deuterium exchange (HDX) rates indicate residues 2200-2228 are a major determinant in G99 binding (24), including K2227 which, upon substitution with glutamic acid, abrogates fVIII binding to G99 (16). The crystal structure of the isolated C2 domain bound to G99 identified a conformational epitope composed of multiple loops and revealed K2227 forms multiple electrostatic contacts with the G99 light chain (20). Here, we present the crystal structure of ET3i (25–27), a bioengineered human/porcine chimera of B domain-deleted fVIII, bound to the G99 antigen binding fragment (F_AB_). Our structure represents the first crystal structure of a fVIII replacement therapeutic bound to an anti-C2 inhibitor, providing insight into how non-classical inhibitors alter the C2 domain conformation of mature fVIII and allosterically influence the thermal motion of nearby epitopes. These results illustrate how fVIII replacement therapeutics can be designed to reduce inhibitor binding and fVIII antigenicity.

## Materials and Methods

### Expression and Purification of ET3i

ET3i was expressed and purified as previously described (25, 26) to a final concentration of 0.8 mg/mL and stored in 50 mM HEPES, pH 7.4, 5 mM CaCl_2_ and 350 mM NaCl at -80°C.

### Purification of G99 F_AB_ Fragments

G99 monoclonal antibodies were expressed and purified in hybridoma cell lines and F_AB_ fragments were prepared as previously described (16, 20). Briefly, large-scale antibody production was performed at the Antibody Production Facility at the Fred Hutch (Seattle, WA). Immunoglobulin (IgG) and F_AB_ purifications were completed with Protein A Plus spin columns and immobilized papain kits (Thermo Scientific, Rockford, IL) according to the manufacturer’s protocols. Purified F_AB_ fragments were stored at a final concentration of 10 mg/mL at -80°C in F_AB_ storage buffer (25 mM Tris-HCl pH 7.2, 100 mM NaCl).

### Crystallization, Data Collection, and Refinement

The ET3i:G99 F_AB_ complex was formed at a 1:1.2 stoichiometric ratio in 50 mM Tris HCl (pH 7.4), 200 mM NaCl, and 2.5 mM CaCl_2_ and purified using a 100 kDa MWCO spin column (Amicon) to 1 mg/mL. Initial crystal conditions were determined *via* high-throughput microbatch crystallization using the Hauptman-Woodward High-Throughput Crystallization Center (Buffalo, NY) (28). Diffraction quality crystals were subsequently grown by hanging drop vapor diffusion in a 1:1 (v/v) ratio of the ET3i:G99 protein complex and crystallization solution containing 50 mM malic acid (pH 7.0) and 8-18% (w/v) PEG 1500, PEG 6000, or PEG 10,000. Crystals were cryoprotected in mother liquor with the stepwise addition of 30% (v/v) glycerol. X-ray diffraction data were collected on the Advanced Light Source (ALS) Berkeley Center for Structural Biology (BCSB) beamline 5.0.1 (Berkeley, CA). Data collection and processing were performed with Adxv, XDS and CCP4 (29). Phasing of the ET3i:G99 crystals was determined with PHASER-MR using a fragment-based molecular replacement approach with the previously determined 3.2 Å structure of ET3i (PDB ID: 6MF0) and the 2.47 Å structure of human factor VIII C2 domain in complex with murine inhibitory antibodies 3E6 and G99 (PDB ID: 4KI5) (20, 27, 30). Model building and refinement were performed with WinCoot and PHENIX, respectively (31). All figures were generated with the PyMOL Molecular Graphics System, Version 2.0 (Schrödinger, LLC).

## Results

### Crystal Structure of Factor VIII in Complex With the Anti-C2 Domain G99 Antibody

**Figure 1:**
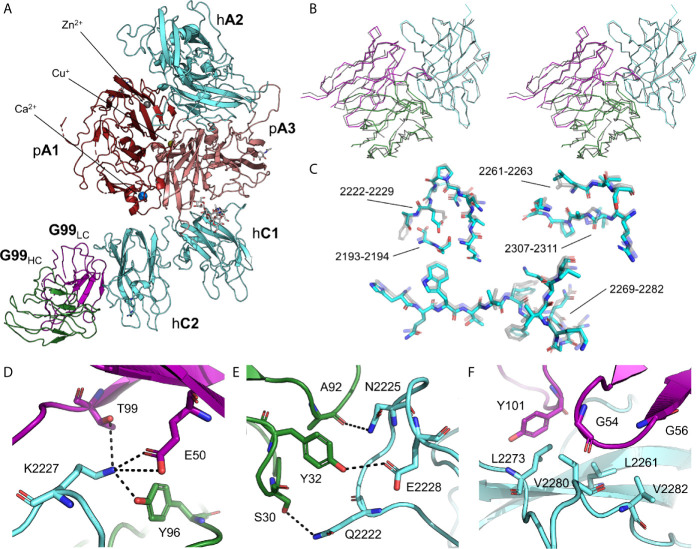
Crystal structure of ET3i bound to the G99 F_AB_ fragment. **(A)** Cartoon representation of the B domain deleted bioengineered fVIII construct (ET3i) bound to the variable domain of G99 inhibitor antibody. Porcine A1 and A3 (pA1 and pA3) domains are colored dark red and pink, respectively, and human A2, C1, and C2 (hA2, hC1, and hC2) domains are colored cyan. Heavy and light chains of the G99 F_AB_ fragment (G99_HC_ and G99_LC_) are colored green and purple, respectively. N-acetylglucosamine modifications are depicted as sticks. **(B)** Ribbon diagram of aligned C2 domain (cyan) and G99 heavy and light chains (green and purple, respectively) from the ET3i:G99 crystal structure and of the isolated C2 domain bound to G99 (grey; PDB ID: 4KI5) in stereo view. **(C)** Stick representation of the G99 epitope from the ET3i:G99 (cyan) and C2:G99 (grey, faded) crystal structures. **(D, E)** Electrostatic contacts between ET3i epitope 2222-2229 (cyan) and G99 heavy chain (green) and light chain (purple). Dotted lines depict hydrogen bonds (distance ≤ 5 Å). **(F)** Hydrophobic residues along the ET3i epitope 2269-2282 (cyan) buried by the G99 light chain (purple).

The X-ray crystal structure of the ET3i B domain-deleted fVIII construct bound to G99 was determined at 4.15 Å resolution and refined to R_work_/R_free_ values of 0.2998/0.3384 (PDB ID: 7KBT) (**Figure 1A** and **Table S1**). The asymmetric unit (ASU) contains one molecule consisting of the A1, A2, A3, C1, and C2 domains of ET3i and the variable domains of the heavy and light chains of G99. While the F_AB_ constant domains were included in the protein complex, these domains could not be modeled into the final structure, presumably due to flexibility, and thus were excluded. The ET3i:G99 complex superimposes well with the crystal structure of the isolated C2 domain bound to G99 (PDB ID: 4KI5) (20) with a root-mean-square deviation (RMSD) value of 0.81 Å^2^ (**Figure 1B**). Both complexes have structurally identical epitopes for the G99 inhibitor antibody which encompass residues 2193-2194, 2222-2229, 2161-2163, 2269-2282, and 2307-2311 (**Figure 1C**). The nature of the C2/G99 binding interface relies on a combination of polar and hydrophobic interactions. **Figure 1D** illustrates how K2227 is a critical residue for binding to G99, participating in multiple electrostatic interactions with E50, T99, and Y96. The epitope spanning residues 2222-2229 provides multiple points of contact with the heavy chain of G99 (**Figure 1E**), including E2228, which has been proposed to interact with the Gla domain of fIXa (23). Lastly, residues L2261, L2273, V2280, and V2282, previously suggested as a binding site for thrombin (22), form direct, extensive contacts with the G99 light chain (**Figure 1F**). Our structure of ET3i bound to the G99 F_AB_ fragment illustrates how non-classical inhibitors potentially block the binding of thrombin, fXa and fIXa, thus preventing dissociation from vWf and formation of the tenase complex.

### Binding G99 Induces a Conformational Rearrangement to the C2 Domain in Mature Factor VIII

**Figure 2:**
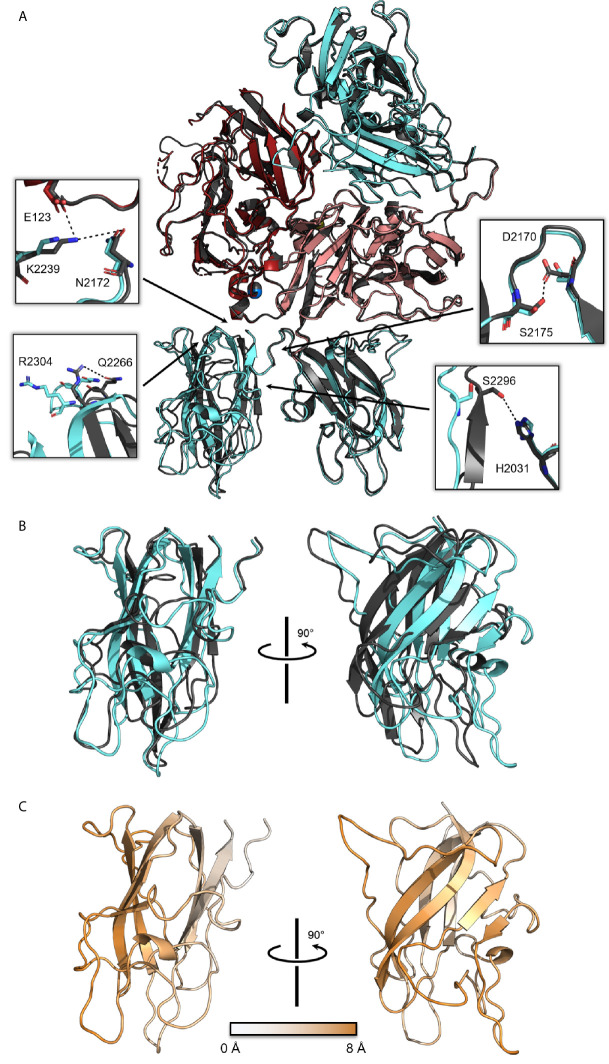
Structural alignment of free ET3i and ET3i:G99 crystal structures. **(A)** Alignment of free ET3i (grey) and ET3i:G99 (A1, dark red; A3, pink; A2/C1/C2, cyan). Insets depict intramolecular contacts that are broken in the ET3i:G99 structure. **(B)** Alignment of the C2 domain from free ET3i structure (grey) and ET3i:G99 structure (cyan). **(C)** Structure of the C2 domain from the ET3i:G99 complex colored as a function of RMSD from alignment with free ET3i. White, low RMSD shifts; orange, large RMSD shifts.

Alignment of the ET3i:G99 complex to the free ET3i structure (PDB ID: 6MF0) (27) suggests that binding G99 does not disrupt the overall ET3i structure, with an average RMSD of 0.68 Å^2^ (**Figure 2A**). The C2 domain, however, shows the largest conformational change upon binding G99 (**Figures 2B, C**), undergoing an ~8 Å translocation relative to the other ET3i domains. Most of these conformational changes are localized to several loops proximal to the G99 epitope, including residues 2256-2265, 2270-2285, and 2305-2314. This rearrangement to the C2 domain is concomitant with breaking multiple interfacial contacts with the adjacent A1 and C1 domains (**Figure 2A**). The A1/C2 interface includes interactions between amino acids E123, N2172, and K2239 that are disrupted due to K2239 shifting by 5 Å in the ET3i:G99 structure. The C1/C2 interface includes interactions between amino acids H2031 and S2296, and D2170 and S2175, both of which become disrupted in the ET3i:G99 complex. Lastly, the ET3i:G99 crystal structure reveals loss of an intramolecular contact within the C2 domain between R2304 and Q2266. While the extent of disruption to these interactions is not definitive given the lower resolution of the crystallographic data, the structure of ET3i:G99 does show significant rearrangement to the C2 domain with the greatest conformational changes occurring to the aforementioned regions.

### Analysis of B-Factor Values From Multiple ET3i Crystal Structures

**Figure 3:**
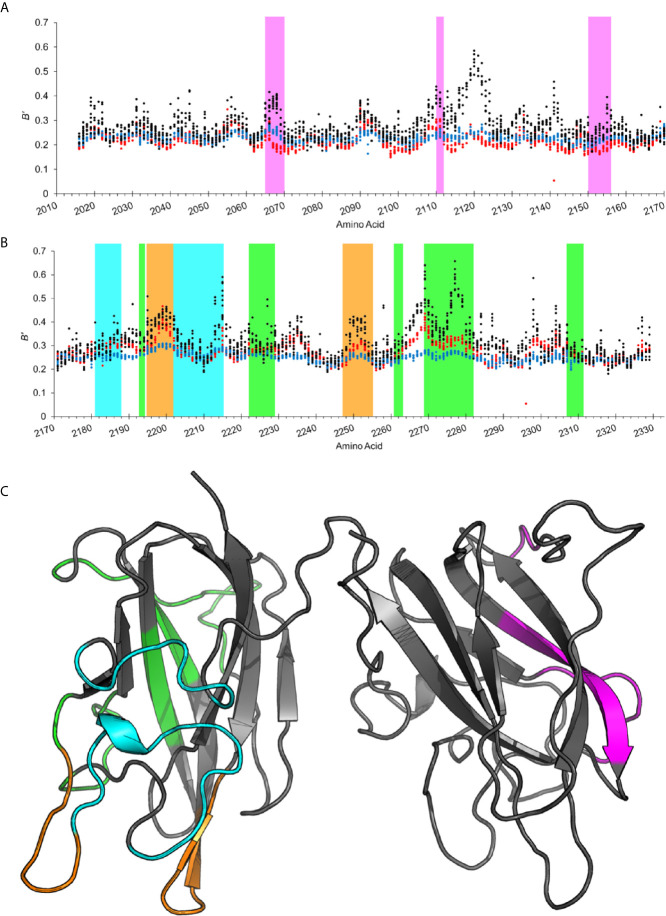
Anti-C domain inhibitors reduce atomic B-factors on solvent-exposed loops. **(A, B)** Normalized atomic B-factors averaged for each epitope in the **(A)** C1 domain and **(B)** C2 domain from free ET3i (black, PDB ID: 6MF0), ET3i:G99 (blue, PDB ID: 7KBT), and ET3i:2A9 (red, PDB ID: 7K66). **(C)** Cartoon representation of the C1 and C2 domains from the free ET3i crystal structure (PDB ID: 6MF0, model A). Antibody epitopes are highlighted (2A9, magenta; G99, green; 3E6, cyan; BO2C11, orange).

To further investigate how inhibitor binding influences the flexibility of C domain epitopes, we performed a comparative B-factor analysis on the C1 and C2 domains from several crystal structures of ET3i bound to antibody inhibitors as well as the free ET3i structure (**Figures 3A, B**). B-factor values are calculated by the spatial fluctuation of atoms in a crystal structure from their equilibrium positions (32) and provide insight into protein structure thermostability, with low values indicating rigidity and high values indicating flexibility (units, Å^2^). In addition to the free ET3i and ET3i:G99 crystal structures, we also included the crystal structure of ET3i complexed with the anti-C1 domain inhibitor 2A9 (PDB ID: 7K66) in our analysis as the C2 domain in this structure undergoes a similar translocation (33). To better compare between each crystal dataset and reduce bias in this study, normalized B-factor values (*B*’) were calculated for each atom using the following equation:

(1) $$B’=\frac{B}{\left(B_{average}\right)\left(Resolution\right)}$$

where *B* is the raw atomic B-factor from the crystal dataset, *B*_average_ is an average of the atomic B-factors of the ET3i molecule in the crystal asymmetric unit, and Resolution is the reported atomic resolution for the respective structure. Model A of the free ET3i crystal structure, which has two ET3i molecules in the ASU (27), was used to compare with the antibody-bound structures.

Atomic *B’* values encompassing the peptide backbone and residues were averaged at known C1 and C2 domain epitopes (**Figure 3C**) from three ET3i crystal structures were averaged and tabulated for comparison (**Table 1**). As expected, regions that experience some of the greatest decreases in *B’* values occur directly at the epitopes from the antibody-bound structures. Residues 2065-2070, 2110-2112, and 2150-2156 in the ET3i:2A9 structure, which encompass the 2A9 epitope, have 35.2%, 27.2%, and 26.1% lower *B’* values, respectively, compared to the free ET3i structure (**Figure S1**). Similarly, residues 2269-2282, which form extensive interactions with the G99 antibody, have a 37.9% lower average *B’* value in the ET3i:G99 complex than the free ET3i structure (**Figure S2**). These observed differences in *B’* values are due to the inhibitory antibody binding to the respective epitope and reducing atomic motions.

**Table 1:** Average *B’* values for C1 and C2 domain epitopes.

	ET3i	ET3i:G99	ET3i:2A9
A1-A2/A3-C1-C2	0.307	0.241 (-21.5%)	0.255 (-16.9%)
**2A9 epitope**			
2065-2070	0.322	**0.247 (-23.5%)**	**0.209 (-35.2%)**
2110-2112	0.352	**0.235 (-33.1%)**	**0.256 (-27.2%)**
2150-2156	0.260	0.218 (-16.4%)	**0.192 (-26.1%)**
**G99 epitope**			
2193-2194	0.319	0.264 (-17.5%)	0.279 (-12.6%)
2222-2229	0.323	0.263 (-18.4%)	0.284 (-12.0%)
2261-2263	0.294	0.241 (-18.0%)	0.260 (-11.5%)
2269-2282	0.422	**0.262 (-37.9%)**	**0.319 (-24.4%)**
2307-2311	0.286	0.239 (-16.3%)	0.266 (-7.1%)
**3E6 epitope**			
2181-2188	0.311	0.257 (-17.4%)	0.293 (-5.9%)
2202-2215	0.326	0.269 (-17.5%)	**0.267 (-18.3%)**
**BO2C11 epitope**			
2195-2202	0.404	**0.291 (-28.0%)**	0.339 (-16.0%)
2247-2255	0.337	**0.251 (-25.5%)**	**0.270 (-19.9%)**

Atomic B’ values were calculated using equation (1) and averaged across the corresponding antibody epitope, including the peptide backbone and side chain. Values in parentheses depict percent differences from the free ET3i structure. Bold values represent differences that are greater than the average ET3i molecule (“coldspots”).

Previous work focusing on the atomic B-factors from crystal structures of the isolated C2 domain bound to classical and non-classical inhibitory antibodies identified fluctuations to the thermostability in certain epitopes (15, 21). We sought to expand our understanding on this topic by calculating the *B’* values in the C1 and C2 domain epitopes when the opposing domain was bound to an inhibitory antibody. Our results indicate that antibody binding is a potent allosteric modulator of atomic motions to adjacent epitope (**Table 1**). For instance, residues 2269-2282, which participate in binding G99 to the C2 domain, have a 24.4% lower average *B’* value in the ET3i:2A9 structure than the free ET3i structure. Similarly, *B’* values from residues that comprise the 2A9 epitope in the ET3i:G99 crystal structure are lower than the free ET3i structure, most notably residues 2065-2070 which have a 23.5% lower average *B’* value. By calculating the average *B’* value for each amino acid at these epitopes, we determined that P2067 and F2068 of residues 2065-2070 and Q2276, N2277, and G2278 of residues 2069-2282 experience the greatest reduction in atomic motions (**Figures S1**, **S2**). None of the aforementioned “coldspots” participate in lattice contacts within the protein crystal, indicating an alternate mechanism for reducing peptide flexibility. While differences in *B’* values can be due to variables that are unaccounted for in equation 1, such as crystallization conditions or refinement strategy, these results are indicative of an allosteric relationship between anti-C1 and anti-C2 epitopes in the presence of inhibitor antibodies.

We next investigated the atomic thermostability for residues spanning the epitopes for 3E6 and BO2C11 antibodies, which bind to unique regions on the C2 domain (**Figure 3C**) and are categorized as classical inhibitors (19–21), in the ET3i:G99 and ET3i:2A9 crystal structures. We measured reduced *B’* values for the 3E6 epitope in the ET3i:2A9 and ET3i:G99 structures when compared with the free ET3i structure. Specifically, the 3E6 epitope spanning residues 2202-2215 had an 18% lower average *B’* in both inhibitor-bound crystal structures (**Table 1**, **Figure S3**). Residues 2195-2202 and 2247-2255, which encompass the BO2C11 epitope (19), have 16% and 19.9% lower *B’* values in the ET3i:2A9 crystal structure, respectively, and are even lower in the ET3i:G99 structure (28.0% and 25.5%, respectively) (**Table 1** and **Figure S4**). Taken together, these results support the hypothesis that these regions on the C2 domain become more rigid when bound to either G99 or 2A9.

## Discussion

In this study, we report on the crystal structure of ET3i, a bioengineered fVIII molecule, bound to the pathogenic, non-classical anti-C2 domain antibody inhibitor G99. While the A domains are structurally unperturbed by G99 binding, the C2 domain undergoes an ~8 Å translocation and loses multiple intramolecular contacts with the adjacent A1 and C1 domains. Mutations at the A1/C2 and C1/C2 domain interfaces have been identified in several hemophilia A cases, including E123K, K2239E, and R2304L/G/C (34–37). Considering the crystal structure of ET3i:G99 indicates binding to G99 disrupts these domain-domain contacts, we speculate how mutations to these regions influence the C2 domain conformation and inhibitor binding. In a previous surface plasmon resonance (SPR)-based study, researchers mapped the epitopes of 11 anti-C2 antibody inhibitors and identified several residues that are not part of a contiguous epitope, yet significantly impact binding to anti-C2 inhibitors when mutated (38). K2239, which participates in multiple hydrogen bonds at the A1/C2 domain interface (**Figure 2A**), demonstrated slightly stronger binding with the classical antibody 3E6 when substituted with alanine. Furthermore, R2304C, which is linked to moderate cases of hemophilia A and high inhibitor titer levels (35, 37), has been proposed to destabilize fVIII without disrupting vWf and phospholipid binding (39). These data are indicative of a unique relationship between C2 domain-domain contacts and fVIII immunogenicity. Disruption to domain interfacial contacts may induce a structural rearrangement to the C2 domain and enhance fVIII immunogenicity. Our structure of the ET3i:G99 complex provides evidence for a connection between disruption to domain-domain contacts near the C2 domain and inhibitor binding.

Our analysis of normalized B-factors from several ET3i structures in the absence and presence of inhibitor antibodies suggests that binding G99 or 2A9 induces structural rigidity on multiple solvent-exposed regions of the C1 and C2 domains that are not part of a contiguous epitope for either antibody. HDX protection patterns have been identified for residues 2231-2252 using the isolated C2 domain bound to G99 (24), despite not forming direct contacts with the antibody. Our results support these findings, with the greatest reduction in atomic motions occurring to residues 2249-2252 at both the amino acid functional group and the peptide backbone (**Figure S2**). We also observed improved thermostability to residues near the A3-C1 domain interface in both inhibitor-bound ET3i crystal structures, most notably residues 2115-2125 and 2137-2146. The A3-C1 domains have previously been shown to contain binding sites for inhibitor antibodies in hemophilia A patient plasmas (40–42), including the patient-derived inhibitor NB41 (43). Reduced flexibility to this region may be due to favorable packing with the A3 domain which could reduce binding to anti-A3-C1 domain inhibitors such as NB41.

Adjustments to the thermostability in certain fVIII epitopes raise important questions regarding immune recognition and cooperativity between classical and non-classical anti-C2 domain inhibitors. Indeed, synthetic peptides encompassing the G99 epitope have been shown to stimulate CD4+ T-cell proliferation and induce an immune response, including residues 2301-2320, which had the strongest response among acquired hemophilia A patients (15). Because the degree of peptide flexibility is a strong determinant in T-cell recognition and binding to T-cell receptors (44–46), reducing the dynamic mobility to certain fVIII epitopes is a potential mechanism in fVIII immune recognition.

Furthermore, group A classical inhibitors have elevated association rates with fVIII when in the presence of non-classical group BC antibodies (47, 48), providing evidence for a positive cooperative immune response to fVIII. Previous structural characterization of the C2 domain bound to G99 and 3E6 F_AB_ fragments supports a polyclonal response to fVIII (20, 49). Our analysis of normalized B-factors suggests that such cooperativity between inhibitors relies on reducing local disorder to these regions. Lowering atomic motions may reduce the conformational diversity of certain epitopes, thereby decreasing the entropic cost in macromolecular association, to provide a high-affinity binding site for antibody inhibitors (21, 50, 51). Modification of these epitopes to prevent rigidification and promote conformational diversity may present a novel strategy in the design of fVIII replacement therapeutics with reduced immunogenicity.

Conversely, there is limited evidence for competition for binding within antibody groups. Inhibitor antibodies BO2C11 and I109 are categorized as group AB inhibitors (16), yet display differential binding mechanisms toward the C2 domain. SPR and x-ray crystallography studies indicate R2215 exclusively binds to BO2C11 (19, 38), yet HDX experiments suggest this region has reduced solvent exposure in the presence of I109 (24). Together, these data could indicate that binding I109 disrupts the conformation of the adjacent BO2C11 epitope, thereby promoting binding to I109. Further research is necessary to investigate negative cooperativity within anti-C2 domain antibody groups.

## Data Availability Statement

The datasets presented in this study can be found in online repositories. The names of the repository/repositories and accession number(s) can be found in the article/**Supplementary Material**.

## Author Contributions

ER planned experiments, performed experiments, analyzed data, and assisted in writing the manuscript. SP, JG, and CW performed experiments and assisted in analyzing data. HS, CD, and PL developed expression and purification procedures for ET3i and G99. PS and KC planned experiments, analyzed data, and wrote the manuscript. All authors contributed to the article and approved the submitted version.

## Funding

The Berkeley Center for Structural Biology is supported in part by the National Institutes of Health, National Institute of General Medical Sciences, and the Howard Hughes Medical Institute. The Advanced Light Source is supported by the Director, Office of Science, Office of Basic Energy Sciences, of the U.S. Department of Energy under Contract No. DE-AC02-05CH11231. The Pilatus detector on 5.0.1. was funded under NIH grant S10OD021832. The ALS-ENABLE beamlines are supported in part by the National Institutes of Health, National Institute of General Medical Sciences, Grant P30 GM124169. This Work Was Supported by the Dreyfus Foundation (Henry Dreyfus Teacher-Scholar Award), the National Science Foundation (MRI 1429164) and the National Institutes of Health/National Heart, Lung and Blood Institute (Award Numbers R15HL103518 and U54HL141981 to PS, Award Numbers R44HL117511, R44HL110448, U54HL112309 and U54HL141981 to CD, HS and PL).

## Conflict of Interest

PL is inventor on a patent application describing ET3i and is an inventor on patents owned by Emory University claiming compositions of matter that include modified fVIII proteins with reduced reactivity with anti-fVIII antibodies. CD, PL and HS are cofounders of Expression Therapeutics and own equity in the company. Expression Therapeutics owns the intellectual property associated with ET3i. The terms of this arrangement have been reviewed and approved by Emory University in accordance with its conflict of interest policies.

The remaining authors declare that the research was conducted in the absence of any commercial or financial relationships that could be construed as a potential conflict of interest.
