# Supplementary material for: Structure of Blood Coagulation Factor VIII in Complex With an Anti-C2 Domain Non-Classical, Pathogenic Antibody Inhibitor
Source: Front Immunol. 2021 Jun 10;12:697602. doi: 10.3389/fimmu.2021.697602 (PMC8223065; doi:10.3389/fimmu.2021.697602)
Supplement: Supplementary file 1 [file DataSheet_1.docx]

Supplementary Material

# Supplementary Figures and Tables

## Supplementary Tables

**Table S1. X-ray data collection and refinement statistics.**

| **Wavelength (Å)** | 1 |
| --- | --- |
| **Resolution range (Å)** | 25.48 - 4.15 (4.298 - 4.15) |
| **Space group** | P 4_1_ 2_1_ 2 |
| **Unit cell** | a = 132.243, b = 132.243, c = 380.029; α, β, γ = 90° |
| **Total reflections** | 52,566 (50,92) |
| **Unique reflections** | 26,284 (2,546) |
| **Multiplicity** | 2.0 (2.0) |
| **Completeness (%)** | 99.08 (98.31) |
| **Mean I/sigma(I)** | 6.31 (0.48) |
| **Wilson B-factor** | 195.91 |
| **R_merge_** | 0.06373 (1.359) |
| **R_meas_** | 0.09012 (1.922) |
| **R_pim_** | 0.06373 (1.359) |
| **CC1/2** | 0.998 (0.373) |
| **CC*** | 1 (0.737) |
| **Reflections used in refinement** | 26,198 (2,504) |
| **Reflections used for R_free_** | 1,987 (191) |
| **R_work_** | 0.2998 (0.4645) |
| **R_free_** | 0.3384 (0.4481) |
| **Number of non-hydrogen atoms** | 11,366 |
| macromolecules | 11,293 |
| ligands | 73 |
| **Protein residues** | 1,444 |
| **RMS (bonds)** | 0.005 |
| **RMS (angles)** | 1.07 |
| **Ramachandran favored (%)** | 78.02 |
| **Ramachandran allowed (%)** | 15.15 |
| **Ramachandran outliers (%)** | 6.83 |
| **Rotamer outliers (%)** | 0.08 |
| **Clashscore** | 15.7 |
| **Average B-factor** | 253.38 |
| macromolecules | 253.25 |
| ligands | 273.82 |

## Supplementary Figures


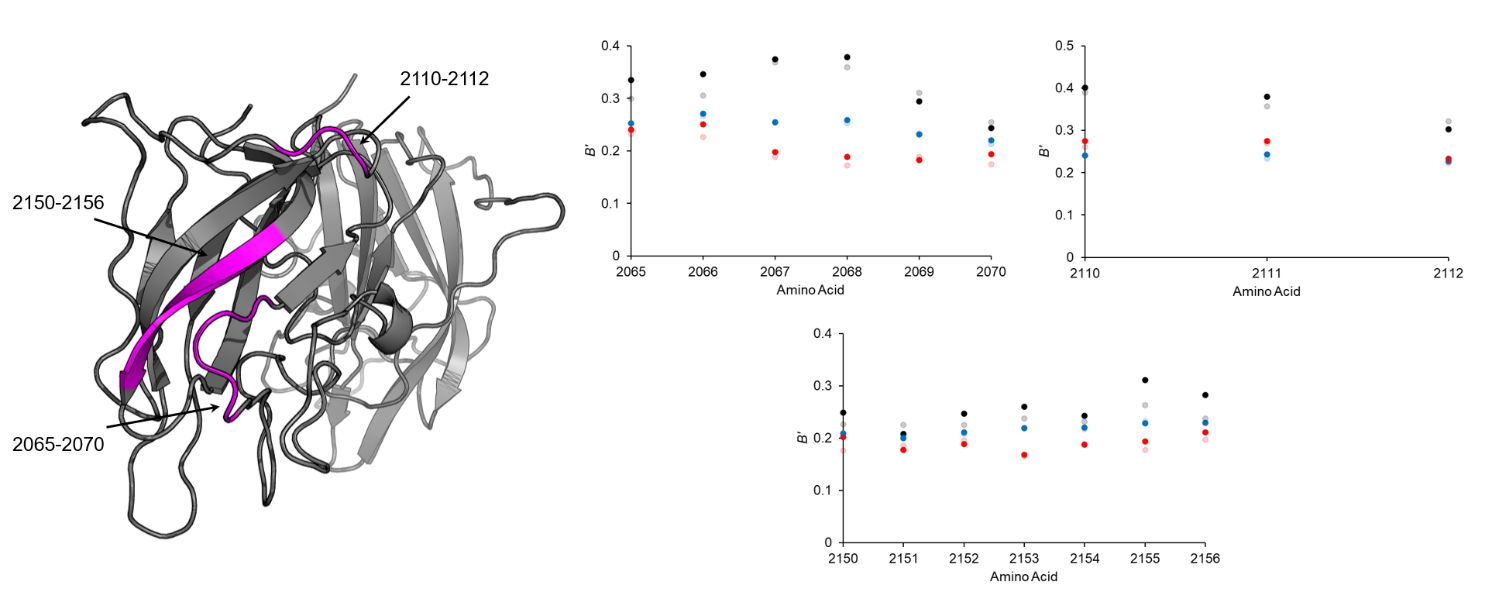


**Supplementary Figure 1.** **Atomic *B’* values per amino acid for 2A9 epitope.** (*Left*) Cartoon structure of the C1 domain highlighting 2A9 epitope (magenta). (*Right*) Solid circles represent the average *B’* of the amino acid backbone and side chain, and faded circles represent the *B’* value of the individual Cα atoms from ET3i (black, PDB ID: 6MF0), ET3i:G99 (blue, PDB ID: 7KBT), and ET3i:2A9 (red, PDB ID: 7K66).


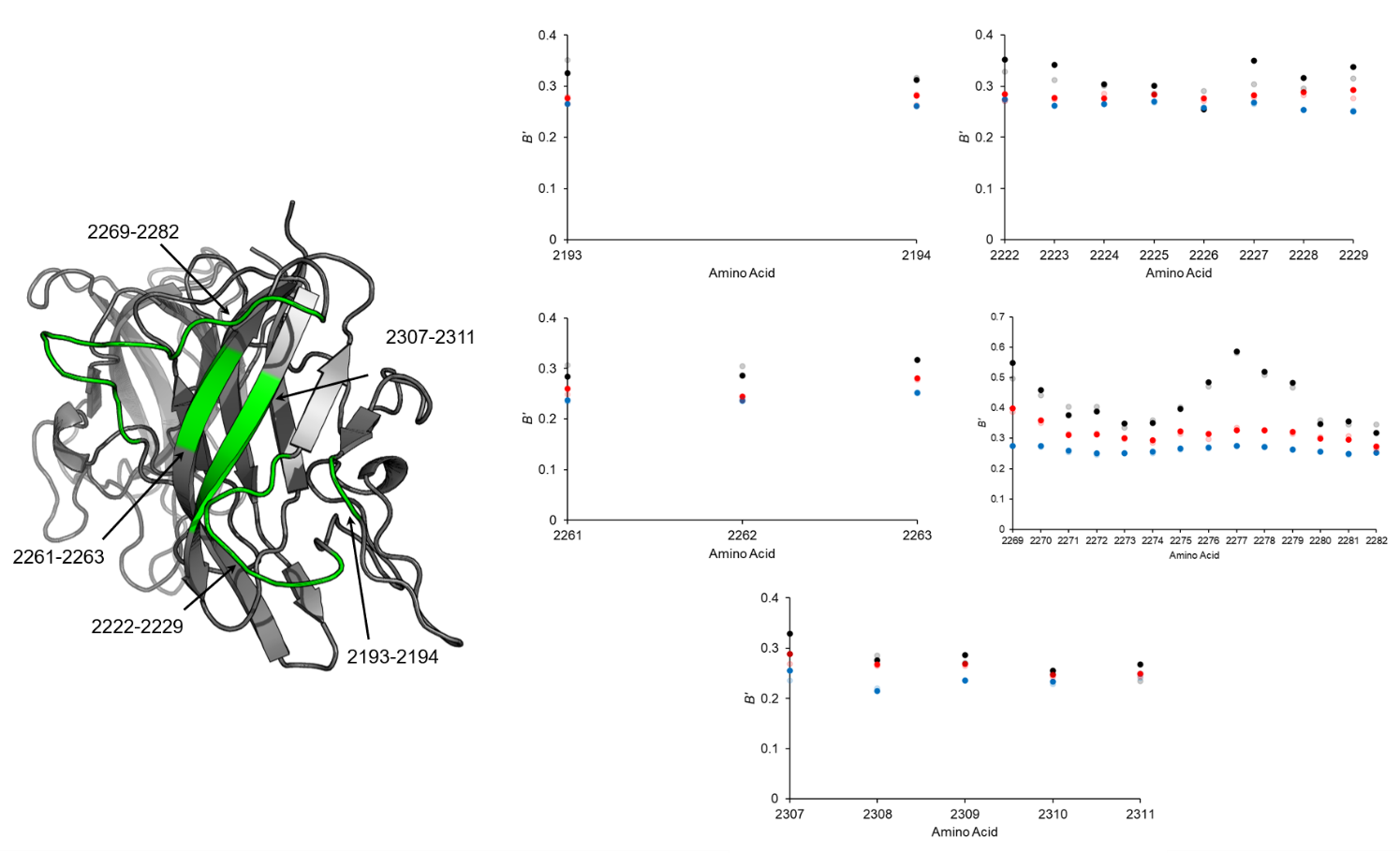


**Supplementary Figure 2.** **Atomic *B’* values per amino acid for G99 epitope.** (*Left*) Cartoon structure of the C2 domain highlighting G99 epitope (green). (*Right*) Solid circles represent the average *B’* of the amino acid backbone and side chain, and faded circles represent the *B’* value of the individual Cα atoms from ET3i (black, PDB ID: 6MF0), ET3i:G99 (blue, PDB ID: 7KBT), and ET3i:2A9 (red, PDB ID: 7K66).


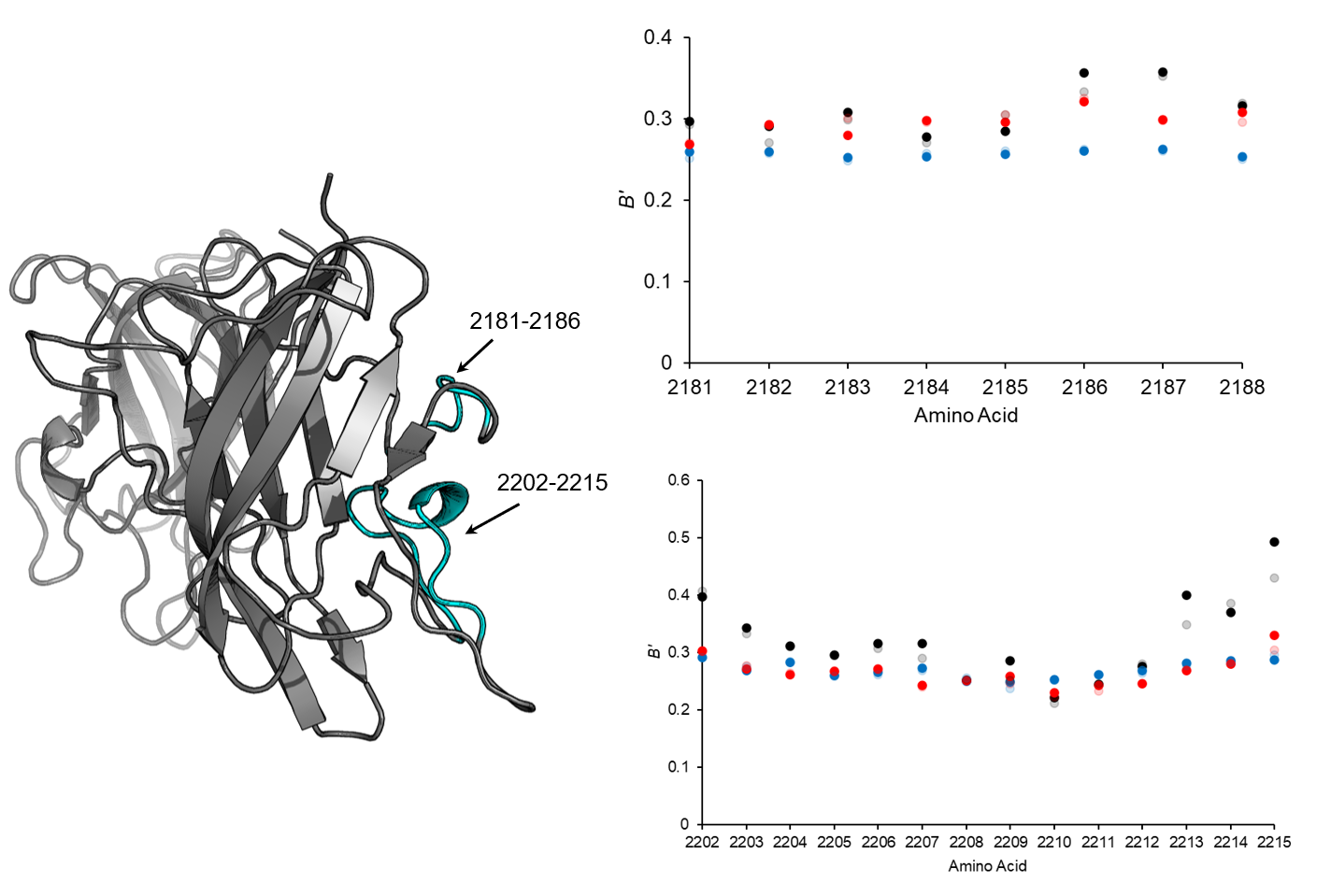


**Supplementary Figure 3.** **Atomic *B’* values per amino acid for 3E6 epitope.** (*Left*) Cartoon structure of the C2 domain highlighting 3E6 epitope (cyan). (*Right*) Solid circles represent the average *B’* of the amino acid backbone and side chain, and faded circles represent the *B’* value of the individual Cα atoms from ET3i (black, PDB ID: 6MF0), ET3i:G99 (blue, PDB ID: 7KBT), and ET3i:2A9 (red, PDB ID: 7K66).


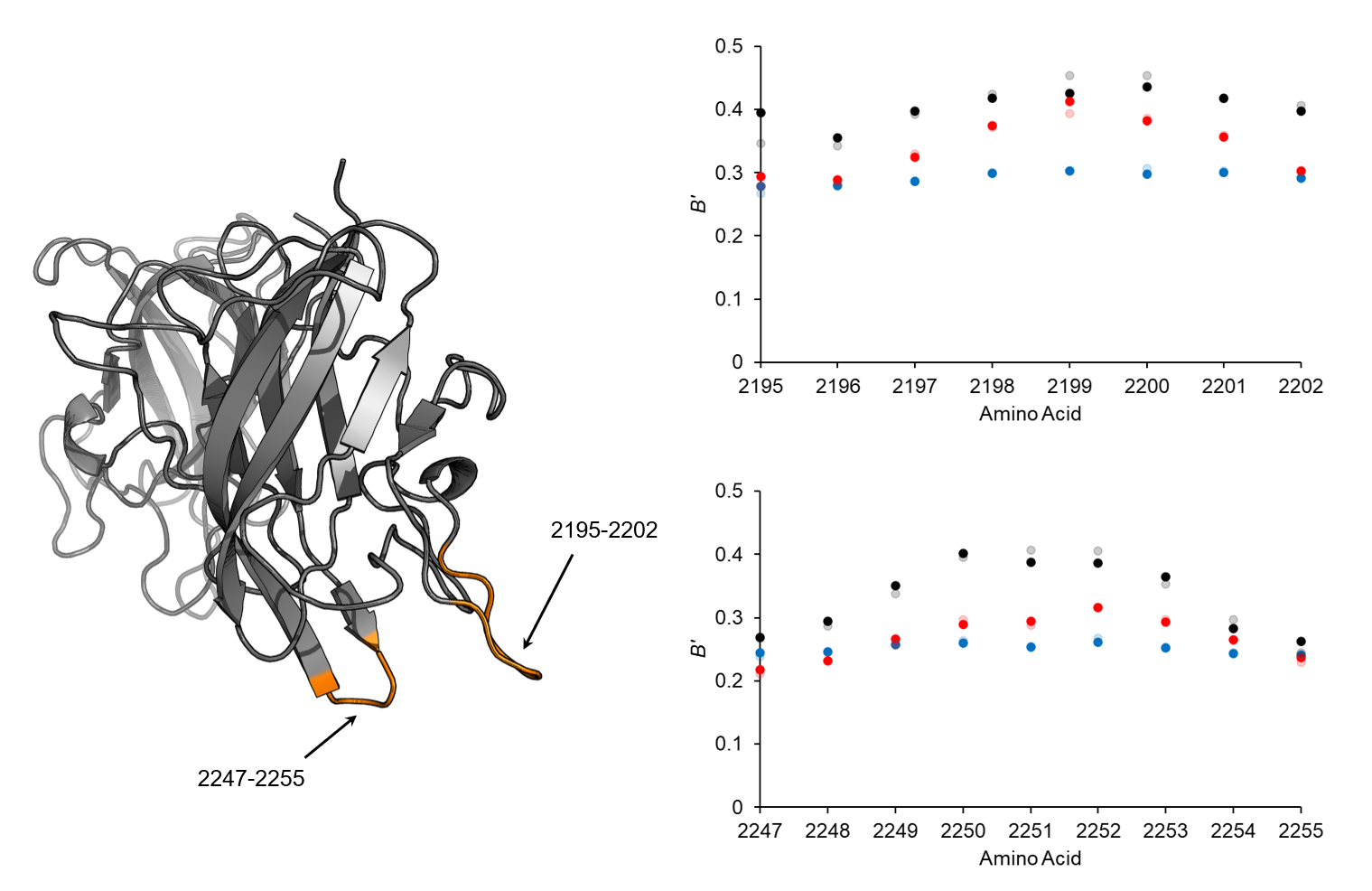


**Supplementary Figure 4.** **Atomic *B’* values per amino acid for BO2C11 epitope.** (*Left*) Cartoon structure of the C2 domain highlighting BO2C11 epitope (orange). (*Right*) Solid circles represent the average *B’* of the amino acid backbone and side chain, and faded circles represent the *B’* value of the individual Cα atoms from ET3i (black, PDB ID: 6MF0), ET3i:G99 (blue, PDB ID: 7KBT), and ET3i:2A9 (red, PDB ID: 7K66).
